# Olfaction and gustation abilities after a total laryngectomy

**DOI:** 10.2478/raon-2013-0070

**Published:** 2014-07-10

**Authors:** Gordana Mumovic, Irena Hocevar-Boltezar

**Affiliations:** 1 University Hospital for Ear, Nose and Throat Diseases, Novi Sad, Serbia; 2 Department of Otorhinolaryngology and Cervicofacial Surgery, University Medical Centre, Ljubljana, Slovenia

**Keywords:** laryngectomy, olfaction, gustation, questionnaire, quality of life

## Abstract

**Background:**

A laryngectomy affects many of a patient’s functions. Besides speech and respiratory-tract problems, olfaction and gustation problems can also have an influence on the quality of life. The aim of this study was to find out how often various nasal problems and decreased gustation appear after a laryngectomy.

**Patients and methods.:**

One hundred and five laryngectomized patients (9 women, 96 men, aged 45–88 years), treated in two tertiary centers, were included in the study. They completed a questionnaire about various nasal problems, olfactory and gustatory capabilities, possible allergies and irritants in their environment, and the impact of the nasal and gustation problems on their quality of life.

**Results:**

Olfaction was impaired in 51.4%, and was even not possible in 30.5%, of patients. Decreased gustation abilities were reported in 26.7%, and dysgeusia in 11.4%, of patients. Almost 21% of patients were bothered by an impaired gustatory ability and 50.5% of patients were affected by their loss of olfaction. Frequent nasal discharge was reported in 20%, frequent sneezing in 58.1%, and nasal itching in 33.3% of the laryngectomized patients. There were no correlations between the age and the olfaction and gustation abilities and between the allergy and the nasal symptoms, whereas the correlation between olfaction and gustation appeared significant (p=0.025).

**Conclusions:**

Various nasal and gustatory problems were reported in more than 80% of laryngectomized patients. The olfaction and gustation abilities are connected and have a substantial impact on the quality of life. Like in the case of speech, the rehabilitation of olfaction is also necessary in all laryngectomized patients and must take place soon after the completion of the treatment.

## Introduction

Laryngectomy is a surgical procedure that is usually reserved for patients with advanced-stage laryngeal or hypopharyngeal carcinoma. It is used when organ-preservation treatment programs are not possible or for the salvage of failure after a non-surgical treatment.^1^ The procedure can cure the patient, but it can also affect many of his/her functions. Respiration and speech are altered forever; swallowing needs to be re-learned; smell and taste are attenuated; lifting heavy objects, straining and coughing are compromised. Therefore, there are numerous potential problems (emotional, psychological, physical, economic, social, and communicative) that can affect the quality of a patient’s life.^2^–^4^

There are a large number of papers on speech and respiratory rehabilitation after a laryngectomy. However, laryngectomized patients have already reported that they do not get enough information about the potential problems with their ability to smell and taste and the consequent changes with respect to eating after the surgical treatment.^5^ However, in the past two decades, olfaction and gustation problems in laryngectomees have begun to receive more attention.

After a laryngectomy, the nose and mouth are disconnected from the lower respiratory tract, which results in a loss of smell. According to a systematic review of the literature dealing with the functions of smell and taste after a laryngectomy, there is a consensus that the inability to make air flow through the nose to reach the olfactory epithelium is the main reason for the deterioration of these functions.^6^ In addition, some authors reported changes in the nasal mucosa and olfactory system that can also affect the ability to smell in laryngectomized patients. Various degrees of neuroepithelial degeneration and decreased proportion of mucus-producing cells were described.^7^,^8^ Other authors reported atrophic nasal mucosa, but normal olfactory mucosa, in the majority of laryngectomized patients.^9^

In a canine model, the olfactory mucosa showed involution after the simulation of a laryngectomy. The changes were supposed to be caused not only by the cessation of the air flow through the olfactory groove, but also by a nonfunctioning connection between the vagal laryngeal innervation and the olfactory cortex.^10^

Veyseller *et al.* performed magnetic resonance imaging in laryngectomees and in subjects with a normal olfactory function, with smaller olfactory bulb volumes found in the former group. However, it is not completely clear whether the lack of sensorial stimulation from the olfactory neuroepithelium in laryngectomized patients and reduced smelling abilities are the only reasons for the decreased size of the olfactory bulb.^11^

In the past two decades there were an increasing number of studies reporting on olfaction and gustation problems after a laryngectomy. Hyposmia or anosmia was reported to be experienced by 35–78 % of patients.^12^–^15^ Gustation was found to be less disturbed after a laryngectomy than odor perception, *i.e.*, in 15% of patients after a laryngectomy.^12^ An interaction between olfaction and gustation was also detected^12^,^13^, but only a few studies report on other nasal problems. *e.g.*, a frequent nasal discharge.^12^

The aim of this study was to find out how often various nasal problems and decreased gustation appear in laryngectomized patients; how these problems influence the quality of their lives; and what are the risk factors for the various affected functions.

## Patients and methods

One hundred and five patients who underwent a laryngectomy for advanced laryngeal or hypopharyngeal cancer in two tertiary centers (University Hospital for Ear, Nose and Throat Diseases, Novi Sad, Serbia and Department of Otorhinolaryngology and Cervicofacial Surgery, University Medical Centre, Ljubljana, Slovenia) more than 6 months previously were included in the study. Sixty-two patients received postoperative or up-front radiotherapy (with the laryngectomy being a salvage procedure). The other patients were treated only surgically. There were 9 women and 96 men, aged 45–88 years (mean 61.86 years, standard deviation 9.45 years). They all completed an anonymous questionnaire about their olfactory and gustatory capabilities. They stated whether they had received radiation therapy or not in the course of the treatment. The patients were asked about various nasal problems (decreased olfaction, nasal discharge, sneezing, and nasal itching) and decreased gustation. They were asked about known allergic diseases before or after the laryngectomy (asthma, allergic rhinitis, allergic dermatitis) and about their living and working environments. They also estimated how much the nasal and gustation problems disturbed them and influenced the quality of their lives. The questionnaire was created for the purpose of the study. The data on the time interval between the laryngectomy and the inclusion of the individual patient in the study, and the details about the radiation therapy given to the patients were not collected.

Analyses were performed using the statistical package SPSS 19.0 (SPSS Corporation, USA). Descriptive analysis and 2-sided t-test, χ^2^-test, Fisher exact test were used and the strength of the correlations between the different parameters were calculated using a Spearman rank correlation. The significance level was set at 0.05.

## Results

The results of the questionnaire about the various nasal symptoms, gustation problems, and factors causing nasal problems are presented in Table 1. There were answers missing in the questionnaires of some patients.

Thirty-seven (35.2%) patients declared that their decreased olfaction abilities bother them and 16 (15.2%) patients stated extreme inconvenience due to a decreased ability to smell. Nineteen (18.1%) patients stated that their decreased or altered ability to taste bothers them and three (2.9%) patients were very affected by it. Four (3.8%) patients declared that their social life was affected because of their nasal discharge.

A decreased or altered gustation ability appeared significantly more often in the patients with decreased olfaction abilities (37 out of 86, 43%) than in the group with the normal ability to smell (3 out of 19, 15.8%) (p = 0.035). Among 40 patients with decreased or altered gustation abilities, there were 37 (92.5%) patients with a decreased or absent olfaction ability. A significant correlation was found between the olfaction and gestation abilities (R_S_ = 0.222, p = 0.025). There were no significant associations between the age and olfaction abilities (R_S_ = 0.090, p = 0.532) and between the age and gustation abilities (R_S_=−0.098, p = 0.511).

Sixty-nine (65.7%) patients stopped working after their laryngectomy and retired. The others did not confirm the possibility of irritants at their living or working place (12, 11.4%) or did not answer the question about possible irritants in their environment (24, 22.8%). Therefore, no analysis with regard to the microclimate in their environment influencing nasal problems was possible.

Among those with known allergic diseases (asthma, allergic rhinitis, allergic dermatitis) there were significantly more patients with nasal discharge compared to the group without allergy (87.5% *vs.* 71.9%, p = 0.035). No other significant differences were detected between these two groups (Table 2).

## Discussion

The results of our study showed that the olfaction and gustatory functions were reduced in a substantial proportion of laryngectomized patients; this affected the quality of their lives. The results also revealed that other nasal problems (nasal discharge, sneezing) were common after a laryngectomy.

In the present study, more than 50% of patients reported a decreased quality of life because of the loss of an ability to smell and/or taste after the laryngectomy. Specifically, among 105 laryngectomized subjects, olfaction was impaired in 51.4% and was even not possible in 30.5% of patients. Decreased gustation abilities were reported in 26.7%, and dysgeusia in 11.4%, of patients. Almost 21% of patients were bothered by an impaired gustatory ability and 50.5% of patients were affected by their loss of olfaction.

Some authors have reported similar results. Caldas *et al.* detected fewer patients with olfaction and gustatory problems after a laryngectomy in their study than were discovered in ours. In their group of 63 laryngectomized patients, 52% of the patients reported hyposmia, while 15% reported dysgeusia. There was a significant correlation between hyposmia and dysgeusia, as was found in our study as well. All the patients with taste problems from Caldas’s study also had a reduced ability to smell.^6^ In our study, 37 out of 40 patients with dysgeusia had a decreased or absent olfaction ability. A significant correlation between hyposmia and dysgeusia was also found in several other studies.^12^,^13^

Smelling can be orthonasal or retronasal. In the first case the odorants come in the nasal cavity and olfactory epithelium through the nostrils. In the latter case the olfactory area is reached from the mouth through the nasopharynx and choanae.^16^ While orthonasal stimuli are strongly associated with sniffing and the airflow through the nostrils, retronasal stimuli are also related to the intake of food.^17^ Chewing induces an air stream into the oral cavity by which the olfactory organ can be stimulated backwards.^18^ Most of the taste sensation depends upon retronasal olfaction.^19^ This can be the reason for fewer gustatory than olfactory problems in our laryngectomized patients.

Smelling and gustation abilities can decrease with advancing age, although no significant correlation between the age and the olfaction abilities was found in the present study.

Other nasal problems in our laryngectomized patients were also identified. A nasal discharge was reported to appear occasionally in 53%, and frequently in 20%, of the included patients. Four patients declared that their social life was affected because of their nasal discharge. Nasal discharge in laryngectomized patients has already been reported in several studies.^12^,^20^,^21^ Sesterhenn *et al.* studied the incidence of sinunasal disease in laryngectomized patients. They stated that sinunasal diseases appear in patients after laryngectomy less frequently than before surgery. In addition, they report on an increased nasal discharge that is not related to common colds, acute or chronic sinusitis.^20^ The reason for the increased mucous production in laryngectomized patients could be the increase of goblet cells, which starts in the second week after the laryngectomy.^22^ Deniz *et al.* also report on a hypersecretory phase in an early period after the laryngectomy.^23^ On the other hand, some authors report on decreased mucus production during the first 12 post-operative months.^8^ In our study we included only those patients who had a laryngectomy at least 6 months before the start of the survey. For this reason, we suppose that the problems with nasal discharge persisted for more than just during the early postlaryngectomy period in our patients. However, because the data on the length of time after the laryngectomy were not included in the anonymous questionnaire we were not able to confirm our hypothesis.

The patients from our study complained about other nasal problems as well, e.g., frequent sneezing in 58.1%, and nasal itching in 33.3%, of cases. As the causes of nasal discharge, sneezing and nasal itching can be allergies or irritants, we tried to determine their importance on the incidence of these symptoms. Unfortunately, about two-thirds of the included patients were retired after the laryngectomy and the others could not assess the presence of irritant substances in their environment. Therefore, the analysis of the impact of irritants in the living or working place on some nasal symptoms could not be performed.

Allergies and some allergic diseases were reported by 16 of the patients. The presence of nasal discharge was the only significant characteristic of the allergic patients in comparison to the others without allergy. There were no significant differences with regard to frequent nasal discharge, type of nasal discharge, sneezing or nasal itching. We concluded that allergy did not significantly influence the nasal symptoms in our patients. The nasal problems can thus be related to the laryngectomy. A possible explanation could be a longer nasal mucociliary clearance time in the laryngecomized patients, which has already been described in some studies^22^, and consequently the persistence of various irritating air-born particles on nasal mucosa in laryngectomized patients.

It was also reported that gustation can change after radiation therapy of the head and neck cancer. A loss of gustation abilities was most pronounced 2 months after the completed radiation therapy and recovered gradually and not always completely during the first year after the treatment.^24^ In a Japanese study including 118 patients irradiated in the head and neck region, a loss of taste was found when the anterior part of the tongue was included in the radiation field.^25^ The main cause of diminished gustation abilities resulting from the radiation therapy is probably the disappearance of taste buds on the tongue.^26^ In our study the questionnaire was anonymous and therefore the data on radiation-therapy details were not known. Thus we cannot exclude the possibility that some of the gustation problems were the consequence of the radiation therapy and not related only to the laryngectomy and the loss of smelling abilities.

Sinkiewicz *et al*. reported that the loss of olfaction abilities was related to the time after the laryngectomy.^27^ As the data on time between the laryngectomy and the entry of every patient into the study were not collected, it was impossible to analyse the time dependence of the occurrence of the impairment of gustation, olfaction and other nasal problems.

A deficiency of the study was also the use of a not-yet-validated tool. The questionnaire was designed for the study with the purpose of including all possible nasal and gustation problems in the laryngectomized patients, their impact on the quality of the patients’ lives and to exclude other possible reasons for the studied problems. In order to increase the impact of the study, the questionnaire should be validated on a larger group of patients and healthy controls, and compared to a validated tool for the assessment of quality of life.

In conclusion, we can say that nasal and gustatory problems are not rare among laryngectomized patients. They are connected and have a substantial impact on the quality of the patients’ lives. Therefore, as in the case of speech, the rehabilitation of olfaction is necessary in all laryngectomized patients and must take place soon after the treatment is completed.

## Figures and Tables

**TABLE 1.** Nasal symptoms, allergic diseases (asthma, allergic rhinitis, allergic dermatitis), olfaction and gustation abilities in laryngectomized patients (N = 105)

**Nasal or gustation problem**		**No. of patients (%)**
Nasal discharge	Never	24 (22.8%)
	Sometimes	56 (53.3%)
	Often	21 (19%)
	The same as before the laryngectomy	2 (1.9%)
Nasal discharge	Not present	24 (22.9%)
	Watery	66 (62.9%)
	Mucous	14 (13.3%)
Often sneezing	No	44 (41.9%)
	Yes	61 (58.1%)
Nasal itching	No	70 (66.7%)
	Sometimes	32 (30.5%)
	Often	2 (1.9%)
Allergy	No	89 (84.8%)
	Yes	16 (15.2%)
Olfaction ability	The same as before the laryngectomy	19 (18.1%)
	Decreased	54 (51.4%)
	Completely absent	32 (30.5%)
Gustation ability	The same as before the laryngectomy	65 (61.9%)
	Decreased	28 (26.7%)
	Altered	12 (11.4%)

**TABLE 2.** Nasal symptoms and gustation problems in the laryngectomized patients with and without known allergic diseases (N = 105)

**Nasal or gustation problem**	**Patients with known allergic diseases N = 16**	**Patients without known allergic diseases N = 89**	**p**
Nasal discharge	14 (87.5%)	64 (71.9%)	0.035
Frequent nasal discharge	4 (25%)	17 (19.1%)	0.482
Frequent sneezing	10 (62.5%)	49 (55.1%)	0.641
Nasal itching	8 (50%)	26 (29.2%)	0.138
Decreased olfaction ability	14 (87.5%)	71 (79.8%)	0.732
Handicap because of loss of olfaction ability	8 (50%)	46 (51.7%)	1.000
Decreased or altered gustation ability	4 (25%)	35 (39.3%)	0.270
Handicap because of loss of gestation ability	2 (12.5%)	24 (26.9%)	0.753
